# Nicotine Induces Podocyte Apoptosis through Increasing Oxidative Stress

**DOI:** 10.1371/journal.pone.0167071

**Published:** 2016-12-01

**Authors:** Xiqian Lan, Rivka Lederman, Judith M. Eng, Seyedeh Shadafarin Marashi Shoshtari, Moin A. Saleem, Ashwani Malhotra, Pravin C. Singhal

**Affiliations:** 1 Renal Molecular Research Laboratory, Feinstein Institute for Medical Research, Hofstra North Shore LIJ Medical School, New York, United States of America; 2 Academic Renal Unit, Southmead Hospital, Bristol, United Kingdom; University of Houston, UNITED STATES

## Abstract

**Background:**

Cigarette smoking plays an important role in the progression of chronic kidney disease (CKD). Nicotine, one of the major components of cigarette smoking, has been demonstrated to increase proliferation of renal mesangial cells. In this study, we examined the effect of nicotine on podocyte injury.

**Methods:**

To determine the expression of nicotinic acetylcholine receptors (nAChR subunits) in podocytes, cDNAs and cell lysate of cultured human podocytes were used for the expression of nAChR mRNAs and proteins, respectively; and mouse renal cortical sections were subjected to immunofluorescant staining. We also studied the effect of nicotine on podocyte nephrin expression, reactive oxygen species (ROS) generation (via DCFDA loading followed by fluorometric analysis), proliferation, and apoptosis (morphologic assays). We evaluated the effect of nicotine on podocyte downstream signaling including phosphorylation of ERK1/2, JNK, and p38 and established causal relationships by using respective inhibitors. We used nAChR antagonists to confirm the role of nicotine on podocyte injury.

**Results:**

Human podocytes displayed robust mRNA and protein expression of nAChR *in vitro* studies. *In vivo* studies, mice renal cortical sections revealed co-localization of nAChRs along with synaptopodin. *In vitro* studies, nephrin expression in podocyte was decreased by nicotine. Nicotine stimulated podocyte ROS generation; nonetheless, antioxidants such as N-acetyl cysteine (NAC) and TEMPOL (superoxide dismutase mimetic agent) inhibited this effect of nicotine. Nicotine did not modulate proliferation but promoted apoptosis in podocytes. Nicotine enhanced podocyte phosphorylation of ERK1/2, JNK, and p38, and their specific inhibitors attenuated nicotine-induced apoptosis. nAChR antagonists significantly suppressed the effects of nicotine on podocyte.

**Conclusions:**

Nicotine induces podocyte apoptosis through ROS generation and associated downstream MAPKs signaling. The present study provides insight into molecular mechanisms involved in smoking associated progression of chronic kidney disease.

## Introduction

It is estimated that there are more than a billion cigarette smokers all over the world, and over one third of them above 15 years of age [1, 2]. Cigarette smoking has been well known critical risk factor for various diseases including lung, cardiovascular, and cancer. Clinical reports have demonstrated that cigarette smoking plays important role in the progression of chronic kidney disease (CKD), and it worsens CKD in patients with diabetes, hypertension, polycystic kidney disease, and post kidney transplant [2–4]. In addition, smoking may cause “de novo” renal injury to those people who are healthy and have no pre-existing CKD [2, 5–12].

Among the thousands of compounds present in the tobacco smoking, nicotine has obtained special attention since it is regarded to be responsible for both the addictive properties of tobacco smoking and a variety of biological effects that may play an important role in the pathogenesis of different conditions [2, 13]. Nicotine plays its effects via the activation of the nicotinic acetylcholine receptors (nAChRs) [2, 11]. Both *in vitro* and *in vivo* studies demonstrated that nAChRs expressed by mesangial cells contribute to the proliferation of mesangial cells in response to stimulation by nicotine [14, 15]. nAChRs also exist in proximal tubular cells, and their interaction with nicotine results in apoptosis or epithelial-mesenchymal transition (EMT) of these cells [16–18].

Podocytes are terminally differentiated and highly specialized epithelial cells in the Bowman’s capsule in the kidneys. They wrap around capillaries of the glomerulus, and extend foot processes to form a blood urine filtration barrier. Most of the proteinuric diseases are associated with podocytopathy (altered podocyte phenotype; reduction in number and effacement of foot processes) [19, 20]. The presence of nAChRs in podocyte has not been evaluated; moreover, the effect of nicotine on podocytes has not been studied yet. In this study, we examined the effect of nicotine podocyte apoptosis and the involved mechanism.

## Materials and Methods

### Animal

FVB/N mice were purchased from Jackson Lab (Bar Harbor, ME, USA), and were housed within the rodent holding facilities in the Feinstein Institute for Medical Research (Northwell Health) in Manhasset, New York. All animal procedures and protocols were approved by the Institutional Animal Care and Use Committee (IACUC, approval #2009–012) at the Feinstein Institute. It is under temperature, light and humidity control. Adequate food, water, and bedding are provided. Two male and two female mice at 8 weeks old were used in this study. Mice were sacrificed by carbon dioxide asphyxiation and death was confirmed by cervical dislocation.

### Reagents

Nicotine, N-acetyl-L-cysteine (NAC), and 2,2,6,6-Tetramethyl-1-piperidinyloxy, free radical, 2,2,6,6-Tetramethylpiperidine 1-oxyl (TEMPO), methyllycaconitine citrate (MLA), mecamylamine hydrochloride (MEC), VAS2870 (VAS), were purchased from Sigma-Aldrich (St. Louis, MO). SP600125 and SB203580 were purchased from Santa Cruz Biotechnology (Dallas, TX), and PD98059 was from Cell Signaling Technology (Danvers, MA).

### Culture of human podocytes

Conditionally immortalized human podocytes were provided by Dr. Moin A. Saleem (Academic Renal Unit, Southmead Hospital, Bristol, UK), and were cultured as discussed in our previous work [21, 22]. Briefly, immortalized human podocytes proliferated in the growth medium containing RPMI 1640 supplemented with 10% fetal bovine serum, 1 X penicillin- streptomycin, 1 mM L-glutamine, and 1 X insulin, transferrin, and selenium (ITS) (Invitrogen, Grand Island, NY) at permissive temperature (33°C). When the cells reached about 80% confluence, they were transferred to 37°C for differentiation in a medium without ITS for 6 days.

Before nicotine treatment, the differentiated human podocytes were cultured in RPMI 1640 medium with 1% FBS for 12 h. Nicotine were added into the same medium, and then were used to treat podocyte.

### RT-PCR

Total RNA was isolated from human podocytes using Trizol reagent (Invitrogen). Five micrograms of total RNA were reverse transcribed using the first-strand synthesis system (Invitrogen). PCR was performed by using Platinum PCR SuperMix High Fidelity (Invitrogen). Sequences of primers for human nAChR subunits were listed in Table 1. GAPDH was used as internal control, and its forward primer was CCATGGAGAAGGCTGGGC, and reverse primer was CAAAGTTGTCATGGATGA. Amplification was performed at 95°C for 5 min, followed by 30 cycles at 94°C for 1 min, 55°C for 30 s, 68°C for 30 s with a final extension cycle for 5 min at 68°C. DNA samples were visualized by 2% agarose gel electrophoresis.

**Table 1:** Primer sequences for Nicotine receptor subunits (nAChRs).

Subunit	Forward primer	Reverse primer
**α2**	ACCAAATGATGACCACCAACG	AGAACAATGTCGGGGATCCAG
**α3**	ATGCTGTGCTGTCCCTCTCTG	ACGATCAATCACCATGGCAAC
**α4**	GGCGTCCAGTACATTGCAGAC	CGGTCCCTTCCTAGATCATGC
**α5**	GGAAGCTGCGCTCGATTCTAT	CAGGAACAAAAAGCCCAAGAGA
**α6**	GCACTCGCCTGAAGTTGAAGA	GCCCTGCAGTTCCAAATACAC
**α7**	GGTGGTGGTGACAGTGATCGT	CTCTTCATTCGCAGGAACCAC
**α9**	CATCCTGTTGGCCATGACTGT	ATGATGGTCAACGCAGTGGAG
**α10**	ACTCAGGCGAGAAGGTGTCG	CACTGGGACCACAGTAATGCAG
**β2**	TTCATCGCAGACCACATGC	AGGGCCTCACTTGGAGCTG
**β3**	CCCCAGAGAAAGAGGAGAGTCA	CCACAGGAAGATTCGGTCAAG
**β4**	GAGGTTCCGACAGGATGTGC	CTGCATGGGTCTGGAAGAGG

### Western blotting analysis

Western blotting was performed using established methodology [22]. Briefly, cells were washed with PBS and lysed in RIPA buffer (1 X PBS, pH7.4, 0.1% SDS, 1% NP-40, 0.5% sodium deoxycholate, 1.0 mM sodium orthovanadate, 10 μl of protease inhibitor cocktail (100 x, Calbiochem) per 1 ml of buffer, and 100 μg/ml PMSF). Proteins (20–30 μg) were separated by 12% SDS-polyacrylamide gel electrophoresis (PAGE) and then transferred on an Immuno-Blot polyvinylidene fluoride (PVDF) membrane (Bio-Rad, Hercules, CA). After blocking in PBS/Tween (0.1%) with 5% nonfat milk, the membrane was incubated with primary antibodies overnight at 4^°^C followed by horseradish peroxidase-conjugated secondary antibodies (Santa Cruz, 1:3000) and then developed using Enhanced Chemiluminescent (ECL) solution (Pierce). Primary antibodies used were rabbit anti-nephrin (Abcam, 1:1000), goat anti-nAChR α5, α6, α7, β3 receptors (Santa Cruz, 1:1000), rabbit anti-cleaved caspase-3 (Cell Signaling, 1:1000), rabbit anti-Bax (Santa Cruz, 1:1000), rabbit anti-Bcl-2 (Santa Cruz, 1:1000), and goat anti-actin (Santa Cruz, 1:3000). For protein expression quantification, the films were scanned with a CanonScan 9950F scanner and the acquired images were then analyzed using the public domain NIH image program (http://rsb.info.nih.gov/nih-image/).

### Immunofluorescent microscopy

Immunofluorescent microscopy was performed as discussed in our previous work [22]. Briefly, the kidneys were perfused in situ and then fixed with fresh 4% PFA and stored at -80°C. Subsequently, paraffin sections (4 μm) were prepared and de-paraffinized in xylene and re-hydrated through graded concentrations of alcohol. Epitope retrieval was carried out by heating the samples at 98°C for 2 h in Retrieveall-1 (Signet Laboratories, Inc.). Subsequently, cooled samples were permeabilized with 0.3% triton X-100 for 10 min, and were blocked with 2% BSA in 0.1% triton X-100 for 1h at room temperature. Sections were then incubated with primary antibodies overnight at 4°C, followed by Alexa Fluor secondary antibodies (Invitrogen, 1:800), donkey anti-rabbit IgG Alexa Fluor 568 or donkey anti-goat lgG Alexa Fluor 488, for 1 hour at room temperature. Primary antibodies included goat anti-nAChR α5, α6, α7, β3 receptors (Santa Cruz, 1:100), rabbit anti-synaptopodin (Santa Cruz, 1:100). All antibodies were diluted in 0.1% Triton X-100, 2% BSA in PBS. Cells were then counterstained with DAPI to identify nuclei (Sigma-Aldrich). Morphological changes were visualized and captured with a ZEISS microscope (Carl Zeiss MicoImaging GmbH, Jena, Germany) equipped with a digital imaging system.

### Ki-67 staining

Human podocytes (5 x 10^4^) were planted in 35 mm dishes, and were differentiated for 6 days before use. After appropriate treatment, immunofluorescent staining was performed as previous report [23]. Briefly, the medium was removed, and the cells where successively fixed with 4% PFA, permeabilized with 0.3% triton X-100, and were blocked with 2% BSA in 0.1% triton X-100. Then, the cells were incubated with primary antibody, rabbit anti-Ki-67 (Santa Cruz, 1:100), overnight at 4°C, followed by Alexa Fluor secondary antibodies (Invitrogen, 1:800), donkey anti-rabbit IgG Alexa Fluor 568 for 1 hour at room temperature. Nuclei were stained with Hoechest 33342. Staining results were visualized and captured with a ZEISS microscope, and Ki-67 positive cells were counted.

### Apoptotic cell determination

We detected apoptotic cells by using Hoechst taining, following former reports [24, 25]. Briefly, after appropriate treatment, the culture media was removed, and the cells were fixed with 4% PFA for 15 min. After that, Hoechst 33342 (10 μg/ml) was added. After 10 min, cell images were taken with a ZEISS microscope (Carl Zeiss MicoImaging GmbH, Jena, Germany) equipped with a digital imaging system. Apoptotic cells were identified as nucleus condensed and fragmented.

### Intracellular ROS measurement

Human podocytes were differentiated in 96-well plates for 6 days as mentioned above, and were then cultured in serum free medium for 12 h. Subsequently, 0.1 to 10 μM nicotine was added. After incubation for another 12 h, intracellular ROS generation was determined by measuring the fluorescence intensity as discussed in our previous work [22].

### Statistical analyses

Data were presented as means ± standard deviation (SD) unless otherwise noted. All experiments were repeated at least three times with duplicate or triplicate samples in each assay. All data were evaluated statistically by the analysis of variance (ANOVA), followed by Nweman-Keuls multiple comparison tests using software (Prism 4.0, GraphPad Software). In the case of single mean comparison, data were analyzed by t test. P values < 0.05 were regarded as statistically significant.

## Results

### Nicotinic acetylcholine receptors are expressed in podocyte

**Fig 1:**
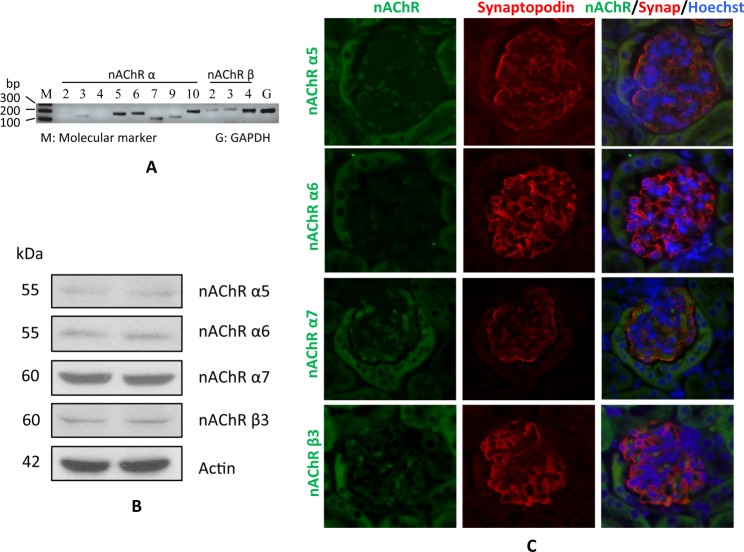
Nicotine receptor subunits (nAChRs) are expressed in podocytes. A. Total RNAs were prepared from differentiated human podocytes, and were used for RT-PCR to detect the expression of nAChR subunits. GAPDH was used as internal control. B. Cell lysate was collected from differentiated human podocytes, and was subjected to Western blotting to detect the expression of nAChRs. C. Paraffin sections were prepared from 2-month-old mice kidneys, and immunofluorescence staining was performed to detect the expression of nAChR subunits. Synaptopodin was used as a marker of podocytes.

Nicotinic acetylcholine receptors (nAChR subunits) have been reported to express in kidney mesangial cells and tubular cells [14–18], but their expression in podocytes has not been studied. Therefore, firstly we examined the expression of nAChR subunits in podocytes. *In vitro* study, we conducted RT-PCR analysis by using human podocytes as the RNA source. The results revealed higher level expression of nAChR α5, α6, α7, α10, and β4 in human podocytes, but the expression of nAChR α3, α9, β2, and β3 were relatively lower (Fig 1A). Meanwhile, we found that the expression of nAChR α2 and α4 were barely detectable (Fig 1A).

We then collected the cell lysate from human podocyte, and performed Western blotting to examine the protein expression of nAChR subunits. Results showed that α7 expressed at a higher level, whereas α5, α6, and β3 expressed at relatively lower levels (Fig 1B).

To confirm this observation *in vivo* studies, we performed immunofluorescence staining for nAChR α5, α6, α7, and β3 in mouse renal cortical sections. Results showed that all these three subunits were highly expressed in glomerular, and they were also partially co-localized with synaptopodin, a podocyte marker (Fig 1C). Combined together, these results demonstrate that podocytes display expression of nicotine receptors.

### Nicotine causes podocyte injury

**Fig 2:**
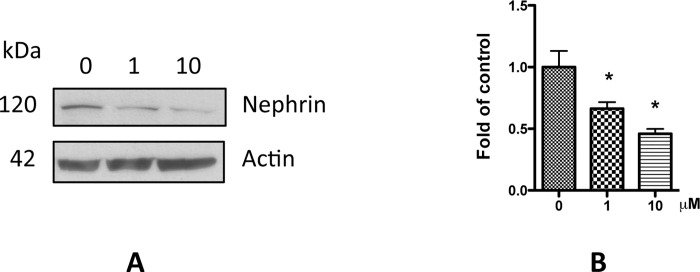
Nicotine decrease nephrin expression in podocyte. Differentiated human podocytes were treated with nicotine (1 and10 μM) for 48 h. Cell lysates were then collected and subjected for Western blotting to detect nephrin expression. A. Representative gels are displayed. B. Quantification of the expression of nephrin in **A,** and the results (mean ± SD) represent three independent samples. * p < 0.05 compared with control (0 μM).

To test whether nicotine causes podocyte injury, we treated human podocytes with 1 and 10 μM nicotine for 48 h, and then collected the cell lysate for Western blotting for evaluation of nephrin expression, one of the most important constituents of slit diaphragm. The results showed that nicotine decreased the expression of nephrin in a dose-dependent manner (Fig 2); these findings confirmed the role of nicotine in the induction of podocyte injury.

### Nicotine doesn't increase podocyte proliferation

Nicotine has been demonstrated to increase the proliferation of renal mesangial cell [4]. To detect whether it has similar function on podocyte, we treated differentiated human podocyte with 0.1, 1, and 10 μM nicotine for 3 days, and then examined the changes of cell numbers by cell counting. Results showed that the total cell numbers didn't significantly change after nicotine treatment (data not shown). To further confirm this observation, we performed immunofluorescent staining to test the changes of Ki-67 positive cell ratio. Results showed that the Ki-67 positive cell ratios among the treatments didn't significantly change (data not shown). Combined together, these indicate that nicotine doesn't promote podocyte proliferation.

### Nicotine increases podocyte apoptosis

**Fig 3:**
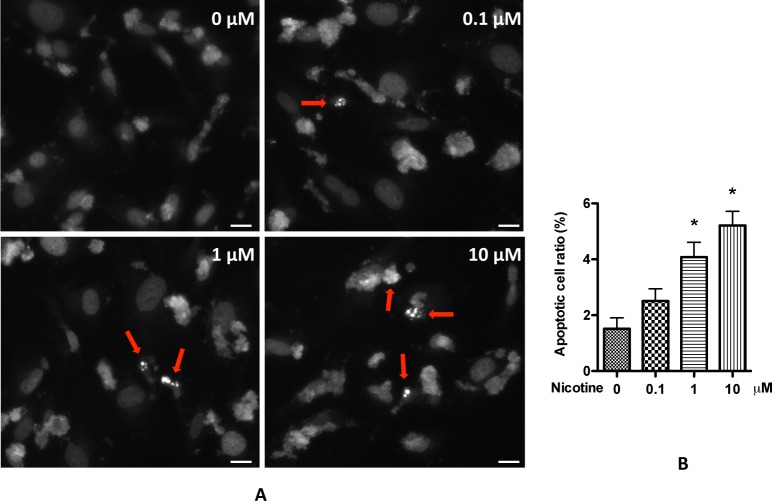
Nicotine induces podocyte apoptosis. Differentiated human podocytes were treated with nicotine (0.1–10 μM) for 48 h. Apoptotic cells were then determined and counted by using Hoechst staining, as describe in Materials and Methods. A. Representative pictures were selected to show the apoptotic cells (red arrow). Scar bar was for 50 μm. B. Results (mean ± SD) were calculated from 20 pictures of each treatment. *, p < 0.05 compared with control (0 μM).

Then, we tested whether nicotine causes apoptosis to podocyte. We treated human podocytes with 0.1, 1, and 10 μM nicotine, and then examined the apoptotic cells by Hoechst staining. We observed that apoptotic cells were barely observed after 24 h, and there was no obvious difference among these treatments (data not shown). However, after 48 h, we observed different apoptotic cell ratios. At 0.1 μM, nicotine increased podocyte apoptosis but the result was not statistically significant; when the concentration reached to 1–10 μM, apoptotic cell ratio dramatically increased. These results indicate that nicotine induces podocyte apoptosis in a dose-dependent manner (Fig 3A and 3B).

**Fig 4:**
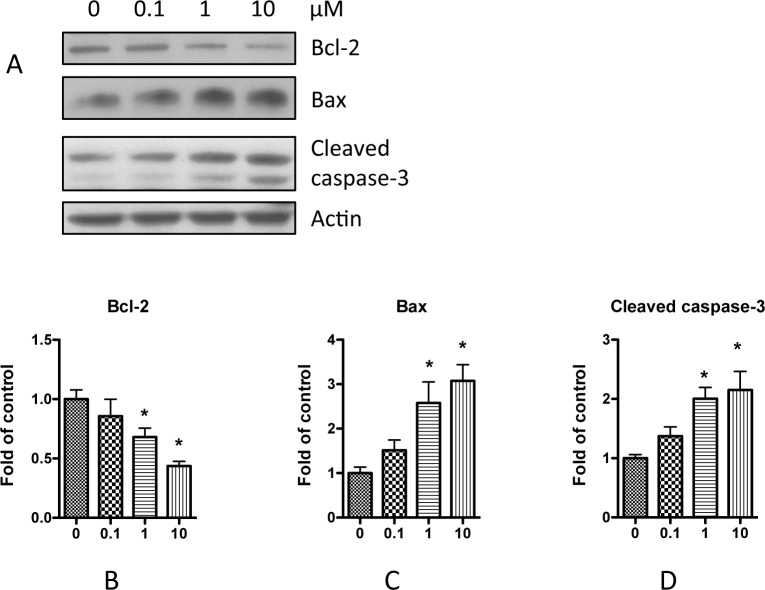
Nicotine treatment affects apoptosis related proteins in podocyte. Differentiated human podocytes were treated with nicotine (0.1–10 μM) for 24 h. Cell lysates were then collect and were subjected for Western blotting. A. Representative results were selected to show the Western blottings. B-D. Quantification of the expression of Bcl-2 (**B**), Bax (**C**), and Cleaved caspase-3 (**D**) in **A,** and the results (mean ± SD) represent three independent samples. * p < 0.05 compared with control (0 μM).

We also examined whether nicotine could affect apoptosis related proteins. Caspase-3 plays the key role in apoptosis, and its cleaved active peptide has been used as the biomarker of apoptosis. To further confirm the effect of nicotine on podocyte apoptosis, we collected the cell lysate after nicotine treatment, and performed Western blotting. Results showed that nicotine increased the cleaved caspase-3 expression (Fig 4). In addition, nicotine increased the expression of Bax, a pro-apoptotic enzyme; conversely, nicotine decreased Bcl-2, an anti-apoptotic enzyme (Fig 4). Taken together, these results clearly demonstrate that nicotine increase podocyte apoptosis.

### Nicotine induces podocyte apoptosis through ROS generation

**Fig 5:**
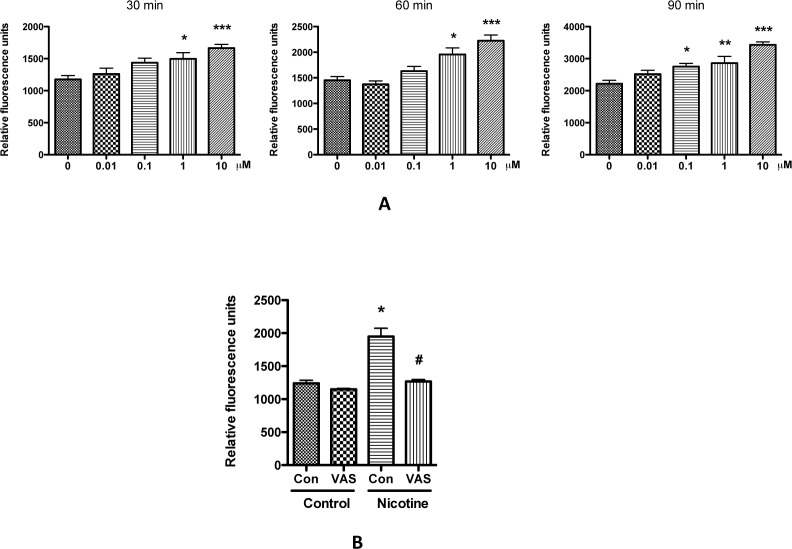
Nicotine increases ROS generation in podocyte. A. Differentiated human podocytes were treated with 0.01 to 10 μM nicotine for 12 h, and were labeled with DCFH for 30 min. After washing with PBS, the cells were incubated at room temperature, and the ROS generation was determined after different periods of time. * p < 0.05, ** p < 0.01, and *** p < 0.001 compared with control (0 μM). B. Differentiated human podocytes were pre-treated with VAS2870 (10 μM) for 1 h, followed by treatment with 10 μM nicotine for another 12 h. Subsequently, the cells were labeled with DCFH and the ROS generation was determined at 1 hour as described above. *p< 0.05 compared with control (0 μM) while #p<0.05 compared with nicotine treatment alone.

Our group and others have demonstrated that ROS is a significant contributing factor for podocyte injury and for the progression of chronic kidney disease [22, 26–28]. ROS has been shown to increase podocyte apoptosis [28–31]. To examine the effect of nicotine on intracellular ROS production, the fluorescence intensity of the intracellular fluoroprobe (DCFH) was evaluated. Results showed that at low concentrations, such as 0.01 and 0.1 μM, nicotine stimulated ROS generation slowly; while, when the concentration reached 1–10 μM, ROS generation was quickly increased when compared with non-treated cells (Fig 5A). Nicotine increased ROS generation in a dose-dependent manner. To detect whether nicotine-induced ROS generation is through the activation of NADPH oxidases, we pre-treated the human podocytes with NADPH oxidase specific inhibitor VAS2870, followed by treatment with nicotine. VAS2870-pretreated podocytes didn't increase ROS generation (Fig 5B), indicating that nicotine increased ROS generation through the activation of NADPH oxidases.

**Fig 6:**
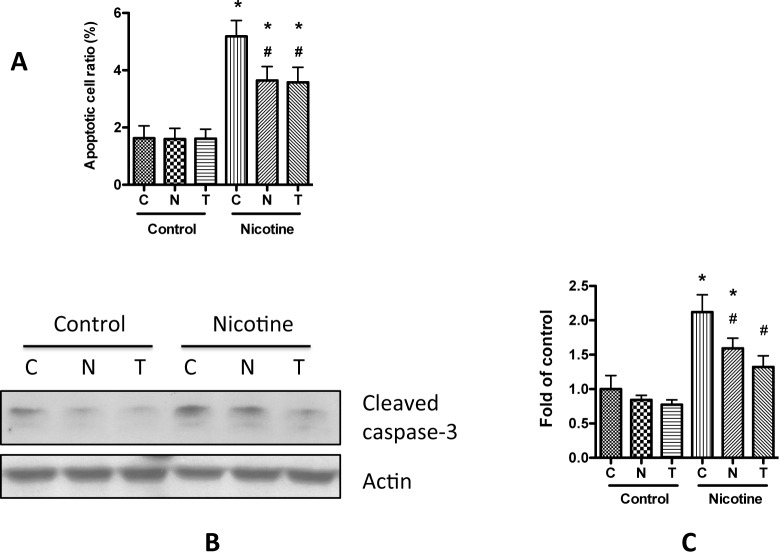
ROS scavengers attenuate nicotine-induced podocyte apoptosis. **A**. Differentiated human podocytes were treated with nicotine (10 μM) for 48 h in the presence or absence of NAC (100 μM) or TEMPO (10 μM). Apoptotic cells were then determined and counted by using Hoechst staining, as described in Materials and Methods. Representative results (mean ± SD) were calculated from 20 pictures of each treatment. **B**. Differentiated human podocytes were pretreated with NAC (100 μM) or TEMPO (10 μM) for 1 h before 10 μM nicotine was added. After another 24 h incubation at 37°C, the cell lysates were collected for Western blotting. **C**. Quantification of the expression of Cleaved caspase-3 in **A,** and the results (mean ± SD) represent three independent samples. * p < 0.05 compared with blank control, while # p < 0.05 compared with nicotine treatment alone. C, control; N, NAC; T, TEMPO.

To determine whether nicotine-induced apoptosis is through ROS generation, the podocytes were pre-treated with ROS scavengers either NAC or TEMPO, and then incubated in media containing 10 μM nicotine. After 24 h, the cells were fixed with PFA, and the apoptotic cells were counted following the Hoechst staining, as mentioned in Materials and Methods. As shown in Fig 6A, pretreatment of the human podocytes with NAC or TEMPO significantly attenuated nicotine-induced podocyte apoptosis. We also performed Western blotting to detect the changes of cleaved caspase-3 expression, and found that addition NAC or TEMPO decreased the its expression (Fig 6B and 6C). Taken together, these data suggest that nicotine-induced ROS generation may be a contributor to the podocyte apoptosis.

### MAPK kinase pathways are involved in nicotine-induced podocyte injury

**Fig 7:**
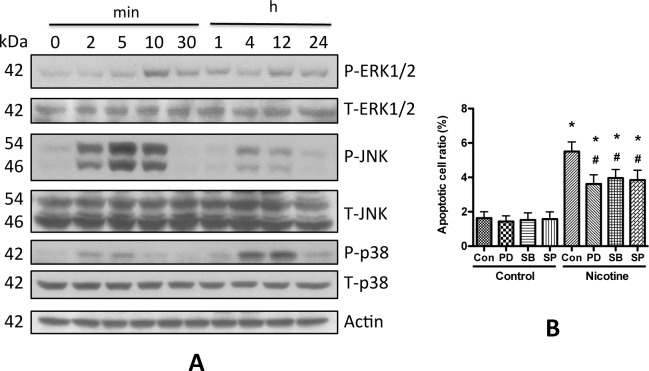
MAPKs regulated nicotine-induced podocyte apoptosis. A. Differentiated Human podocytes were starved in serum free medium for 12 h, and then 0.1 μM nicotine was added. Cell lysates were collected at different time points, and Western blotting was performed to detect the phosphor-ERK1/2, JNK, and p38. Total proteins and actin were used as loading control. **B**. Human podocytes were pretreated with of PD98059 (PD, 5 μM), SB203580 (SB, 3 μM), or SP600125 (SP, 5 μM) for 1 h before 10 μM nicotine was added. After incubation at 37°C for another 48 h, apoptotic cells were then determined and counted by using Hoechst staining. Results (mean ± SD) were calculated from 20 pictures of each treatment. * p < 0.05 compared with blank control, while # p < 0.05 compared with morphine treatment alone.

MAPK kinases, including JNK, ERK1/2 and p38 have been implicated in podocyte injury and the progression of chronic kidney diseases (CKD) [32–36]. To examine the involvement of these kinases and factors in nicotine-induced podocyte apoptosis, we first evaluated the phosphorylation of these proteins. We treated human podocytes with 0.1 μM nicotine, and collected the cell lysates at different time points for Western blotting studies. Results showed that nicotine stimulation significantly activated ERK1/2, JNK, and p38 at early time points (Fig 7A). We repeated this experiment with 1 or 10 μM nicotine, and obtained similar results, but the extents of phosphorylation were stronger (data not shown).

To examine the role of activation of MAPK kinases in nicotine-induced apoptosis, apoptotic cell ratios of podocyte were measured after the treatment with nicotine in the presence or absence of SP600125, an inhibitor of JNK, PD98059, an inhibitor of ERK1/2, or SB203580, an inhibitor of p38. As presented in Fig 7B, all these inhibitors partially attenuated nicotine-induced apoptosis. These results indicate that JNK, ERK1/2 and p38 pathways are involved in the regulation of nicotine-induced podocyte apoptosis.

### nAChR α7 subunit plays an important role in nicotine-induced podocyte injury

**Fig 8:**
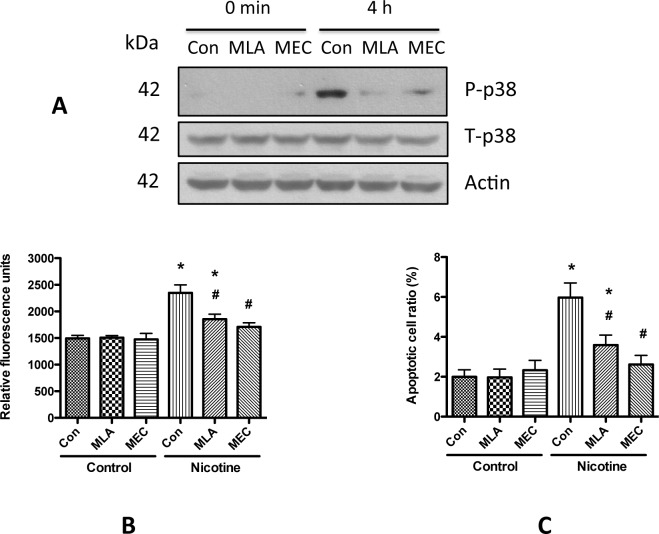
Nicotinic receptor antagonists play important roles in nicotine-induced podocyte injury. A. Differentiated human podocytes were starved in serum free medium for 12 h, pre-treated with MLA (20 μM) or MEC (100 μM) for 1 h, followed by treatment with 10 μM nicotine for another 4 h. Subsequently, the cell lysate was collected for Western blotting to detect the phosphorylation of p38. Total p38 and actin were used as internal control. B. Differentiated human podocytes were pre-treated with MLA (20 μM) or MEC (100 μM) for 1 h, and then were treated with 10 μM nicotine for another 12 h. The cells were labeled with DCFH and the ROS generation was determined at 1 hour as described above. * p < 0.05 compared with blank control, while # p < 0.05 compared with morphine treatment alone. C. Differentiated human podocytes were pre-treated with MLA (20 μM) or MEC (100 μM) for 1 h, followed by treatment with 10 μM nicotine. After incubation at 37°C for another 48 h, Hoechst staining was carried for morphologic assay for apoptotic cells. Results (mean ± SD) were calculated from 20 images for each treatment. * p < 0.05 compared with blank control, while # p < 0.05 compared with morphine treatment alone.

To determine the role of nAChR α7 in the nicotine-induced podocyte injury, we pre-treated human podocytes with either MLA (a nAChR α7 specific antagonist) or MEC (a non-specific nicotinic acetylcholine receptor antagonist) followed by treatment with nicotine. We found that both antagonists significantly blocked nicotine-induced phosphorylation of p38 (Fig 8A). Interestingly, MEC could completely block nicotine-induced ROS generation as well as induction of apoptosis; on the other hand, MLA blocked these effects partially but significantly (Fig 8B and 8C). These results suggest that nAChR α7 plays an important role in nicotine-induced podocyte injury, and other receptor subunits may also be involved.

## Discussion

Podocytes play a vital role in the prevention of glomerular protein leakage during physiological and pathological processes through formation of slit diaphragm [37–40]. Clinical reports have demonstrated that smoking worsens the chronic kidney diseases, and enhances proteinuria [2–12]. Therefore, it is likely that contents of the tobacco smoke directly affect the podocytes. In this study, analysis of RT-PCR, Western blotting, and immunofluorescent staining revealed the expression of several nAChR subunits by podocytes. Nicotine decreased nephrin expression in podocytes, indicating that it caused cell injury to these cells. Nicotine enhanced podocyte oxidative stress resulting into their apoptosis. Nicotine-induced podocyte apoptosis was regulated by the activation of the stress kinase pathways including JNK, ERK, and p38. To our knowledge, this is the first report highlighting the effect of nicotine on podocyte injury.

Nicotine mediates its effects via the activation of muscle and neuronal nicotinic acetylcholine receptors (nAChRs), which are composed of five subunits and expressed by neuronal as well non-neuronal cells [2, 11]. Jaimes *et al* demonstrated the presence of the nAChRs subunits α4, α5, α7, β2, β3 and β4 in human mesangial cells [4], and Kim *et al* reported that nAChRs subunits α3, α5, and β1 are expressed in renal proximal tubular epithelial cells (HK-2) [18]. In this study, we found that the mRNAs of subunits α3, α5, α6, α7, α10, β2, β3 and β4 are expressed in human podocytes. Western blotting and immunofluorescent studies displayed that at least 4 types of these subunits including, α5, α6, α7, and β3 are expressed in podocytes. The different expression profiles of the nAChRs subunits may induce cell dependent effects in response to nicotine types of renal cells. For example, 6 nAChRs subunits are expressed in mesangial cells, but only α4, α7 and β4 contributed to nicotine-induced proliferation, and amongst these subunits, α7 subunit played a major role [4]. Similarly, the subunit α7 subunit has been identified as one of the most important for several of the cholinergic actions mediated by nAChRs in macrophages, vascular smooth muscle cells and cancer cell lines [2, 41, 42]. In the present study, we also found that blocking α7 subunit with MLA significantly attenuated nicotine-induced p38 phosphorylation, ROS generation, and cell apoptosis, indicating that this subunit also plays an important role in nicotine-mediated podocyte injury. However, MLA could not completely suppress nicotine-induced ROS generation and apoptosis, suggesting that other subunits may also be involved; this aspect worth investigating in future studies.

Nicotine has been demonstrated to increase proliferation of renal mesangial cell as well as several other cell types [4, 43–46]. In this study, we treated human podocytes with nicotine for 3 days, but didn’t observe obvious changes on the cell numbers; and immunofluorescent staining results showed that Ki-67 positive cell ratios didn’t change after nicotine treatment. These results indicate that nicotine doesn’t cause podocyte proliferation. Nicotine has also been reported to induce apoptosis in various cells [18, 47, 48]. Consistent with these reports, we found that nicotine increased the number of apoptotic cells and enhanced the expression of apoptotic protein markers including cleaved caspase-3. In addition, nicotine increased the expression of pro-apoptotic protein Bax and decreased the anti-apoptotic protein Bcl-2. These results strongly suggest that nicotine induces podocyte apoptosis.

Nicotine induces ROS generation in a variety of cells and it contributes to the net oxidative stress imposed by cigarette smoking [4, 16, 18, 49, 50]. In previous reported studies, nicotine has been demonstrated to increase the production of ROS generation in culture mesangial cells and stimulated their proliferation and fibronectin production [4, 15]. In this study, we found that nicotine treatment of human podocyte caused a dose-dependent increase in ROS generation, but it didn’t stimulate the proliferation. On the other hand, the increased ROS generation promoted apoptosis in human podocytes, and this effect of nicotine could be attenuated by NAC and TEMPO. Notably, ROS has been incriminated for apoptosis in multiple instances [28–31]. These findings indicate that podocytes behave differently from mesangial cells in nicotine milieu. One possible reason is that podocytes are highly differentiated epithelial cells, and are more vulnerable to increased oxidative stress when compared with mesangial cells, which robustly proliferate on exogenous stimulation. Nicotine also increases the ROS generation in proximal tubule cells, but the consequences are controversial. Arany *et al* reported that nicotine potentiate the effects of TGF-β on α-SMA, vimentin and fibronectin production in proximal tubule cells, indicating that nicotine promotes epithelial-mesenchymal transition (EMT) of these cells [16–17]. On the other hand, recently Kim *et al* reported that nicotine induced apoptosis to renal proximal tubular cells (HK-2 cells) [18]. It's worth noting that in both studies, they used 200–400 μM of nicotine, which is much higher than the peak concentrations found in the plasma of active smokers [51].

Investigating the kinase or transcription factor pathways involved in nicotine-induced kidney injury may provide insight into new potential targets for therapy. Mitogen-activated protein (MAP) kinases, including ERK1/2, JNK, and p38, have been implicated in podocyte injury and the progression of chronic kidney diseases (CKD) [32, 33, 52–54]. All these kinases or transcription factors may also be activated by nicotine in various cells [18, 55–57]. We examined the effect of nicotine on the activation of these kinases and factors in podocytes. Our results revealed that nicotine stimulated the phosphorylation of ERK1/2, JNK and p38. Activation of these pathways has been reported to cause podocyte injury including apoptosis, while suppression of them helps to improve the injury [22, 58–60]. Consistent with these reports, in this study we observed that blockade of these kinases with their specific inhibitors significantly reduced nicotine-induced podocyte apoptosis. Recently Kim *et al* reported that in renal proximal tubular cells (HK-2 cells), nicotine-induced oxidative stress enhanced the phosphorylation of the ERK and JNK signaling pathways, which resulted in the activation of NF-κB signaling pathway and led to apoptosis [18]. In our study, whether the activation of NF-κB signaling pathway is involved in nicotine-induced podocyte apoptosis needs to be investigated in future studies.

Recently, some atypical *N*-methyl-D-aspartate (NMDA) receptors have attracted the interest of researchers for the involved mechanism in the development of nephropathy [61–64]. These NMDA receptors are expressed throughout the kidney, and sustained activation of these receptors in podocytes contributes to oxidative stress, loss of slit diaphragm proteins, and apoptosis [61, 62]. All these effects are similar to nicotinic receptors as described in our study. In addition, the activation of NMDA receptors induces Ca^2+^ influx via cation channels [61, 62], which can lead to glomerulosclerosis. Since several of the nicotinic receptors assembled by the subunits presenting in podocytes are highly calcium permeable [65–71], we speculate that the podocyte nicontine receptors may also have this function. All these findings indicate that nicotinic receptors may cause parallel effects in podocytes akin to transduction mechanisms manifested by NMDA receptors.

In conclusion, we have demonstrated that nicotine has the potential to directly threaten the survival of podocytes, which would contribute to chronic kidney injury. The effects of nicotine are mediated through the generation of ROS, and are regulated by JNK, ERK1/2, and p38 pathways. Our study provides insight into new mechanisms involved in nicotine-induced podocyte damage, and highlights some new therapeutic targets for smoking induced kidney injury.
